# Incorruptible Teeth

**Published:** 1846-03

**Authors:** 


*Incorruptible Teeth.*—At the last meeting of the American Institute, held in the city of New York, a GOLD MEDAL was awarded to Dr. James Alcock for the best specimens of incorruptible teeth, exhibited on the occasion. The teeth manufactured by Dr. A. are now becoming too well known to require such recommendation, to secure for them the confidence of the profession.

